# Visceral leishmaniasis in 26 HIV-negative adults

**DOI:** 10.1186/1756-0500-4-389

**Published:** 2011-10-08

**Authors:** Hazar Lahlou, Ahmed B Filali, Meryem Alami, Moncef Amrani, Rhizlane Berrady, Samira Rabhi, Wafaa Bono

**Affiliations:** 1 Laboratory of Hematology, University Hospital of Fez, Atlas, Fez, Morocco; 2 Internal Medicine Department, University Hospital of Fez, Atlas, Fez, Morocco

**Keywords:** visceral leishmaniasis, HIV-negative adults, bone marrow aspirate, immunofluorescent antibody test

## Abstract

**Background:**

Visceral leishmaniasis is a notifiable parasitic disease that had increased in incidence in our region on the past few years. It is common in children. In adults, it occurs more on a background of immunodeficiency, and frequently with incomplete clinical manifestations, making the diagnosis complicated.

**Findings:**

The aim of our study is to reveal different features of visceral leishmaniasis in adults, through the analysis of its epidemiological, clinical and biological parameters, in a group of 26 patients. No one was infected with HIV or under immunosuppressive therapy Clinical presentation was generally conservative, but there was few differences in adults compared to children, concerning both the clinical symptoms and the laboratory parameters. Diagnosis was provided by direct examination of bone marrow smears in 24 cases (sensitivity 92%), and anti-leishmanial serology in the others.

**Conclusion:**

We should think to the diagnosis of VL even if the patient is not known immunocompromised, and even if the clinical is incomplete, to avoid a delay of care which can lead to serious complications.

## Introduction

Visceral leishmaniasis (VL), also known as kala-azar, is a systemic parasitic infection caused by two of the species of the protozoan *Leishmania *: *L. donovani *and *L. Infantum*. It is spread by the bite of the female sand fly *Phlebotomus *[1]. It affects approximately 500.000 people every year [2-4], and almost 1000 individuals in the Mediterranean basin [5].

Its typical presentation associate fever, splenomegaly, hepatomegaly, lymph node enlargement and pancytopenia.

Conventional laboratory techniques for the diagnosis of VL are parasitological methods, based on the search for parasites on bone marrow smears or other tissues, and in vitro cultivation, and serological methods, favoured by the presence of serum antibodies; the most commonly used are immunofluorescent antibody test (IFAT) and immunoenzymatic methods (ELISA). PCR assays are also proposed as useful tools for the diagnosis of VL, through the detection of *Leishmania *DNA in different biological samples such as peripheral blood and bone marrow aspirate [1,4].

Through this retrospective analysis, we try to discuss the specifics of VL in immunocompetent adults. Patients included are beyond 15 years-old, collected in the internal medicine department at the University Hospital of Fez, between June 2003 and August 2010. All of them were not infected with HIV, and they all underwent a bone marrow aspiration and a serological test of leishmaniasis based on immunofluorescent antibody test (IFAT) for the diagnosis.

## Findings

We collected 26 cases of visceral leishmaniasis diagnosed in HIV-negative adults. Patients ranged in age from 15 to 65 years, with mean ages of 28 years. There were 15 men and 11 women. All patients were from regions already known to be endemic foci of VL, 61% of them were from rural areas, mainly from the vicinity of Fez and Taounate wich are cities in the center of Morocco.

Two patients were diabetic, one had chronic hepatitis C, another suffered from chronic renal failure and in two cases, VL was associated with pregnancy. We have also noted the particular association of VL with pernicious anemia in a woman, and with a spondylarthropathy in an adolescent.

The average consultation time was 107 days. More than half of our patients had consulted the first time for abdominal symptoms. In other cases, hematological and general symptoms were inaugural.

Clinical features of the 26 patients are summarized in Table 1. 84% of our patients had fever, 81% had asthenia and all of them had pallor. Splenomegaly was present in 84% of cases but hepatomegaly and lymph node enlargement were noted in only 35%. Respiratory signs were rare.

**Table 1:** Clinical characteristics of the 26 patients

Clinical data	No. of patients	Percentage
Fever	24	92
Sweats	22	84
Weight loss	11	42
Asthenia	21	81
Pallor	26	100
Haemorrhage	9	35
Splenomegaly	22	84
Hepatomegaly	9	35
Lymphadenopathy	9	35
Edema of lower limbs +/- ascites	2	7
Respiratory signs	3	11
Skin lesions	0	0

Biologically (table 2), anemia was constant, non-regenerative, often normochromic and normocytic, with an average hemoglobin of 7,51 g/dl; It was below 7 g/dl in 40% of cases. Leukopenia was also frequently observed in 76% of cases and thrombocytopenia was less common. In 53% there was a severe neutropenia (< 1000 cells/mm^3^). 11 patients had a platelet count less than 100.000/mm^3^, 7 others had moderate thrombocytopenia (between 100.000 and 130.000/mm^3^), and 8 patients had normal levels of platelets. The rate of serum protein was elevated in 27% of cases, with an extreme of 120 g/l. Most patients had an obvious inflammatory syndrome; in 63% of them, the erythrocyte sedimentation rate (ESR) exceeded 100 mm in the first hour and C-reactive protein (CRP) was often above 50 mg/l and the average value was 62,4 mg/L. only 10 patients had at admission elevated liver enzymes levels, reaching more than 3 times the normal value.

**Table 2:** Biological characteristics of the 26 patients

Parameter	Average value	Minimum value	Maximum value
Hemoglobin (g/dL)	7,51	5,1	10,5
White blood cells (cells/mm^3^)	3208	900	9400
Neutrophils (cells/mm^3^)	1524	380	7300
Lymphocytes (cells/mm^3^)	1207	110	3500
Platelets (10^3^cells/mm^3^)	131	34	394
ESR (mm/h)	99,63	19	150
CRP (mg/L)	62,4	5	219
Total protein (g/L)	81,7	62	120
ALAT (IU/L)	69,32	4	573
ASAT (IU/L)	80,72	5	674

**Figure 1:**
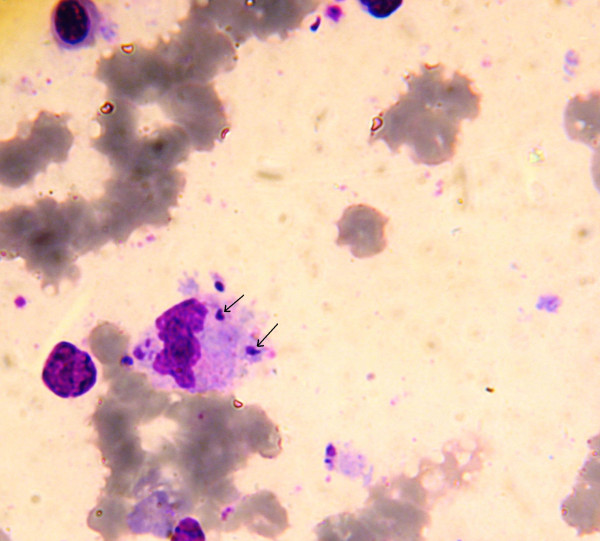
***Leishmania *amastigotes in intracellular, revealed in a Giemsa-stained bone marrow smears**. Amastigotes of *Leishmania *are spherical to ovoid and measure 1-5 μm long by 1-2 μm wide. They possess a large nucleus, a prominent kinetoplast, and a short axoneme, the last of which is rarely visible by light microscopy.

Anti-*Leishmania *serology was performed in 13 patients by IFAT. It was positive in 12 of them. The analysis of bone marrow smears, stained with May-Grunwald-Giemsa, objectified the presence of *Leishmania *amastigotes in 24 cases. The parasites were usually scattered in the stroma in extracellular. Rarely they regrouped in clusters, or were found inside the granulocyte (Figure 1). In 2 cases, the myelogram was normal and no *Leishmania *were demonstrated, probably because of low parasitemia; the diagnosis was therefore established on the basis of clinical and serological data.

An anti-HIV serology was performed in all patients and was negative. It was the same for the serology for hepatitis B and syphilis, made in 15 cases. One patient was positive for Hepatitis C tests.

All patients were treated with injections of antimoniate of Méglumine (Glucantime^®^). They received a single cure of 21 days at a dose of 80 mg/kg/day, administered gradually. This treatment was generally well tolerated. We have noted a single case of liver toxicity that resolved after dose reduction of Glucantime^®^. There was no relapse in our series.

The outcome was favorable in 23 cases (88%), with a progressive improvement in clinical parameters with obtaining apyrexia, disappearing of splenomegaly and improving general condition, then in biological assessments with decreasing of the inflammatory syndrome and normalization of the liver enzymes levels. No infectious or hemorrhagic complications were recorded. Although, the outcome in both pregnant women was characterized by the occurrence of portal hypertension in one, and fetal death in the other. We recorded an unexplained death of a patient of 60 years-old with renal failure, on the 4th day of treatment.

### Epidemiological and etiopathogenic features

During the last years, a modification of the epidemiological profile of VL was noted. This disease is, up to now, quite rare among adults, became increasingly common, especially among immunocompromised individuals. In North-Mediterranean countries for example, there was an inversion of VL case distribution according to age [6,7], in relation to the spread of HIV infection mainly, and also to the use of immunosuppressive therapies, such as long-term corticosteroid treatments [8] and immunosuppressants in systemic diseases, chemotherapy for cancer, and transplant anti-rejection therapy in allograft patients [9]. Any condition able to weaken immune defenses of the host represents a fertile background for the development of VL. This infection is therefore, currently considered as an opportunistic disease. Indeed, several factors have been reported by various authors in literature [10-13], namely diabetes, terminal chronic renal failure, liver cirrhosis, tuberculosis, cancer, and also pregnancy on account of hormonal and immune changes which occur in pregnant women [14].

Nevertheless, several cases of VL were diagnosed in apparently immunocompetent adults, especially in North Africa, with significant proportions in most series. They were around 57%, 45% and 40% according to Afrit et al [11], Aoun et al [10] and Hakem et al [12] respectively.

In our series, the HIV serology was negative each time it was performed. None of our patients had received immunosuppressive therapy, and only 8 cases of VL had occurred on weakened immune terrain (diabetes, pregnancy, renal failure, hepatitis C, megaloblastic anemia and spondylarthropathy). Probably and for the others, it's possible that virulent parasite strains have been selected and may induce disorders even in adults [15].

### Clinical presentation

Clinical manifestations of VL in adults are sometimes discreet and are not always suggestive. Moreover, even when a febrile hepatosplenic pancytopenic syndrome is present, diagnosis is often delayed because those signs are common to several other diseases [16] whose incidence is higher in adults.

**Figure 2:**
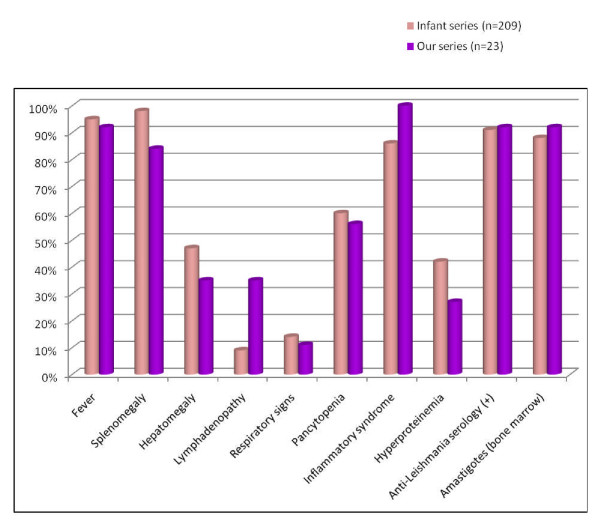
**Comparison of clinical and laboratory features of VL in adults and in children (through an infant VL series about 209 cases published by the pediatric department of our university hospital)**.

Our series included young adults (average age = 28 years) whose clinical manifestations were nonspecific, represented mainly by fever, splenomegaly and anemic syndrome. In comparison to an infant VL series (Figure 2) about 209 cases published by the pediatric department of the same hospital [17], we deduce that clinical presentation in our patients is generally conservative, and the incidence of different symptoms is similar to that of child, except for lymph node hypertrophy, clearly more common in adults, this has been noted also in other mediterranean series. Splenomegaly was present in 84% of cases, but contrary to the child, it was usually moderate. Our result corresponds to that found in another Moroccan series [18]. In other ones [10,11], this sign was absent in almost half the cases. But in the large series of 64 cases of Mediterranean VL in HIV-negative adults reported by Pagliano et al [19], all patients had hepatosplenomegaly (Table 3). Finally, anemic syndrome seems to be the most constant symptom of VL in adults.

**Table 3:** Comparison of different VL series in adults

Parameter (%)	**I. Benlbaghdadi **[18] (n = 6)	**K. Aoun **[10] (n = 18)	**M. Afrit **[11] (n = 7)	**P. Pagliano **[19] (n = 64)	Our series (n = 26)
Fever	100	83	100	94	**92**
Splenomegaly	83	55	57	100	**84**
Hepatomegaly	50	38	57	100	**35**
Pallor	100	94			**100**
Haemorrhage	17				**35**
Lymphadenopathy		33	14		**35**
Pancytopenia	83	22	57		**56**
Inflammatory syndrome	100	83	71		**100**
Hyperproteinemia	50				**27**
Hypergammaglobulinemia	100	94	57		
Anti-*Leishmania *serology (+)	83	82	83	100	**92**
Amastigotes on bone marrow smears	50	67	100	97	**92**
Favorable outcome	83		71		**88**

Deaths	1 case (Hematemesis: PHT)		1 case (Severe sepsis)	1 case (Relapse)	**1 case** **(unexplained)**

PHT = Portal hypertension

### Nonspecific biological signs

Hemogram, erythrocyte sedimentation rate and electrophoresis of plasmatic proteins are the main tests in the diagnosis process in VL. They show non-specific disorders such the common anemia which is normochromic, normocytic and non-regenerative. First moderate, then it gets progressively worse during the evolution, but not as fast as in children. Leukopenia, which was absent in 24% of our patients is, according to other authors, regularly observed [20]. Thrombocytopenia is often delayed or may even miss [21], but late in disease progression, it is sometimes associated with alteration in the synthesis of coagulation factors by the liver, causing bleeding which can be serious or even fatal.

Inflammation is very marked; in fact, ESR that usually reaches 50-80 mm the first hour in children [22], is even higher among adults (99 mm in our series). CRP is also greatly increased.

Total serum protein is highly variable; it may be normal or high, particularly in adults. However, electrophoresis of proteins is very disturbed almost steadily. It shows a polyclonal hypergammaglobulinemia affecting mainly IgG, with hypoalbuminemia [23].

### Specific biological signs

As clinical presentation of VL and hemato-biochemical disruption are unspecific, the detection of serum antibodies by serology, or better yet, the visualization of the parasite in any involved organ, are required for the diagnosis [24].

The anti-*Leishmania *antibodies are detected routinely by direct immunofluorescence technique, ELISA or indirect hemagglutination, and currently by immunoblotting on nitrocellulose membrane [25,26]. Serology can be negative, despite the wealth of the bone marrow in *Leishmania*, especially in immunodeficient patients [6].

PCR also can be used; it may detect *Leishmania *DNA in different biological samples such as peripheral blood and bone marrow aspirate [4].

Concerning parasitological methods, the search for parasites must be first in the bone marrow, and an initial failure does not challenge the diagnosis. In immunocompromised patients, we can even find *Leishmania *on the peripheral blood after leukoconcentration.

In vitro cultivation has a good sensitivity but is carried out only in specialized centers, and if is negative or if the parasites are scanty, the results can be obtained only after several weeks.

In our series, the identification of the parasite by direct examination of the bone marrow allowed to the diagnosis in 24 patients, which means a sensitivity of 92%, comparable to that reported in the literature [27-29]. For the 2 patients whose bone marrow was negative, the diagnosis of VL was selected on the results of serology and clinical manifestations.

## Conclusion

VL affects more and more adults in the Mediterranean area. This means that special care must be given to diagnosis, especially since clinical signs are often incomplete and little characteristic.

Thus, the disease should be suspected whenever there is prolonged or unexplained fever, splenomegaly or hypergammaglobulinemia in a patient from an endemic area, even if he is not infected with HIV or receiving immunosuppressive therapy. And when VL is diagnosed in those adults, an underlying condition that could weaken the immunity of the patient must be seeked, but even if there is not, this does not rule out the diagnosis of VL.

## Competing interests

The authors declare that they have no competing interests.

## Authors' contributions

HL coordinated between the Internal Medicine Department and the Laboratory of Hematology, and drafted the manuscript. AFB performed the statistical analysis. MA prepared and processed images. MAH conceived the study and participated in the literature search. GB operated patient records. SR did a literature search. WB participated in the design of the study and helped to draft the manuscript. All authors read and approved the final manuscript.
